# Distinct mutational backgrounds and clonal architectures implicated prognostic discrepancies in small-cell carcinomas of the esophagus and lung

**DOI:** 10.1038/s41419-021-03754-0

**Published:** 2021-05-12

**Authors:** Zhengbo Song, Yueping Liu, Guoping Cheng, Lianpeng Chang, Zicheng Yu, Ming Chen, Gang Chen

**Affiliations:** 1 Department of Clinical Trial, The Cancer Hospital of the University of Chinese Academy of Sciences (Zhejiang Cancer Hospital), Institute of Basic Medicine and Cancer (IBMC), Chinese Academy of Sciences, Hangzhou, Zhejiang 310022 China; 2 Department of Pathology, Hebei Cancer Hospital, Shijiazhuang, Hebei 050200 China; 3 Department of Pathology, The Cancer Hospital of the University of Chinese Academy of Sciences (Zhejiang Cancer Hospital), Institute of Basic Medicine and Cancer (IBMC), Chinese Academy of Sciences, Hangzhou, Zhejiang 310022 China; 4 Geneplus-Beijing Institute, Beijing, 102206 China; 5 Department of Radiotherapy, The Cancer Hospital of the University of Chinese Academy of Sciences (Zhejiang Cancer Hospital), Institute of Basic Medicine and Cancer (IBMC), Chinese Academy of Sciences, Hangzhou, Zhejiang 310022 China; 6 Department of Pathology, Fujian Medical University Cancer Hospital and Fujian Cancer Hospital, Fuzhou, 350001 China

**Keywords:** Oesophageal cancer, Small-cell lung cancer

## Abstract

Small-cell carcinoma of the esophagus (SCCE) is a rare and aggressive cancer. Although several consistent genomic changes were observed previously between SCCE and small-cell lung cancer (SCLC), detailed mutational landscapes revealing discrepancies in genetic underpinnings of tumorigenesis between these two cancers are scarce, and little attention has been paid to answer whether these genetic alterations were related to the prognosis. Herein by performing whole-exome sequencing of 48 SCCE and 64 SCLC tumor samples, respectively we have shown that the number of driver mutations in SCCE was significantly lower than in SCLC (*p* = 0.0042). In SCCE, 46% of recurrent driver mutations were clonal, which occurred at an early stage during tumorigenesis, while 16 driver mutations were found clonal in SCLC. *NOTCH1/3*, *PIK3CA*, and *ATM* were specifically clonal in SCCE, while *TP53* was clonal in SCLC. The total number of clonal mutations differed between two cancers and presented lower in SCCE compared to SCLC (*p* = 0.0036). Moreover, overall survival (OS) was shorter in patients with higher numbers of clonal mutations for both cancers. In summary, SCCE showed distinct mutational background and clonal architecture compared with SCLC. Organ-specific clonal events revealed different molecular mechanisms underlying tumorigenesis, tumor development, patients’ prognosis, and possible variations in therapeutic outcomes to candidate treatments.

## Introduction

Small-cell carcinoma of the esophagus (SCCE) is a rare and aggressive carcinoma with a poor prognosis, accounting for only 0.6–2.8% of all esophageal malignant tumors^1,2^. Without being covered by National Comprehensive Cancer Network Clinical Practice Guidelines (NCCN Guidelines) in Oncology or Chinese Society of Clinical Oncology diagnosis and treatment guidelines, SCCE is often treated by referring to the guidelines and clinical experience of small-cell lung cancer (SCLC). Nevertheless, inaccurate classification and the substantial genetic heterogeneity across cancer types always lead to decreased therapeutic benefits in SCCE compared with SCLC, resulting in dismal prognosis in patients^3^. Previous study provided a comprehensive genomic profile of SCCE and showed consistent somatic genomic alterations between SCCE and SCLC^4^; however, little attention was paid to the difference in genomic characteristics, and how these disparities might influence therapeutic outcomes.

Occurrence and relative timings of driver mutations lead to the tumor molecular and phenotypic heterogeneities, which lead to variable clinicopathological characteristics and treatment outcomes^5,6^. Small-cell carcinoma originated from different histological lesions, had both convergent and organ-specific mutational patterns, and was demonstrated to undergo highly heterogenous evolutionary processes^7^. Whether the clonal architectures, consisting of clonal driver events (occurring at an early stage during tumorigenesis and found in the majority of tumor cells) and subclonal driver events (occurring at a relatively late stage and found in the small proportion of tumor cells) were consistent between SCCE and SCLC, and whether they are associated with patients’ prognosis remain elusive.

To characterize the clonal architectures and compare the genetic underpinnings of SCCE and SCLC, we performed whole-exome sequencing (WES) of 48 SCCE and 64 SCLC tumor samples, respectively, and analyzed how these genetic features and molecular heterogeneities might affect the clinical outcome in patients.

## Results

### Patient enrollment characteristics

The details of the clinicopathological characteristics of patients enrolled in this study are listed in Table 1. The SCCE cohort was comprised of 48 patients, with a mean age of 61.9 years (range, 46–79 yrs). The median follow-up of the SCCE cohort was 11.1 months (range, 4.0–58.2 months), with 15 (31.3%) patients lost in the follow-up, and the median overall survival was 11.0 months (range, 4.0–21.5 months). The SCLC cohort consisted of 60 males and 4 females, with a mean age of 62 years (range, 43–84 yrs). Most of SCLC patients (93.75%, 60/64) had a history of smoking. The median follow-up of the SCLC cohort was 12.4 months (range, 3.3–113.2 months), with 2 (3.1%) patients lost to follow-up, and the median overall survival was 11.7 months (range, 3.3–49.6 months). No significant association was observed between disease stage or smoking history with patients’ overall survival in SCLC.

**Table 1:** Clinical characteristics of patients with SCLC and SCCE.

Patients	SCCE (*n* = 48)	SCLC (*n* = 64)
	No. (%)	No. (%)
*Gender*		
Male	34 (73%)	60 (94%)
Female	12 (26%)	4 (6%)
NA	2	
*Age at diagnosis*		
Median	62	63.5
Range	46–79	43–84
*Stage*		
I	3 (6%)	3 (5%)
II	11 (23%)	6 (8%)
III	16 (33%)	18 (29%)
IV	12 (25%)	35 (55%)
NA	6 (13%)	2 (3%)
*Smoking history*		
Smoker	31 (65%)	60 (94%)
Non-smoker	14 (29%)	4 (6%)
NA	3 (6%)	0

### WES sequencing analysis

**Fig. 1:**
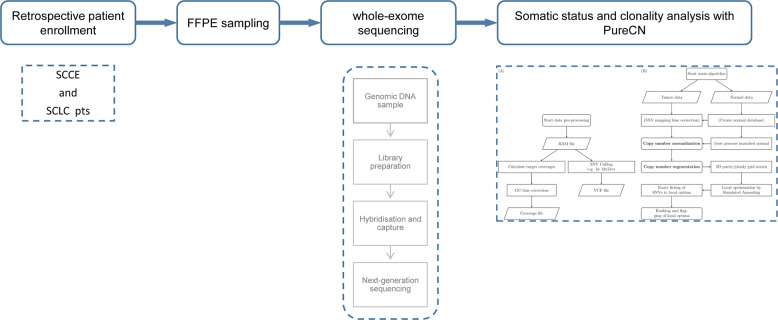
Study design and pipeline. FFPE of patients with SCCE and SCLC was sequenced using WES. Somatic mutations were evaluated with Pure CN.

**Fig. 2:**
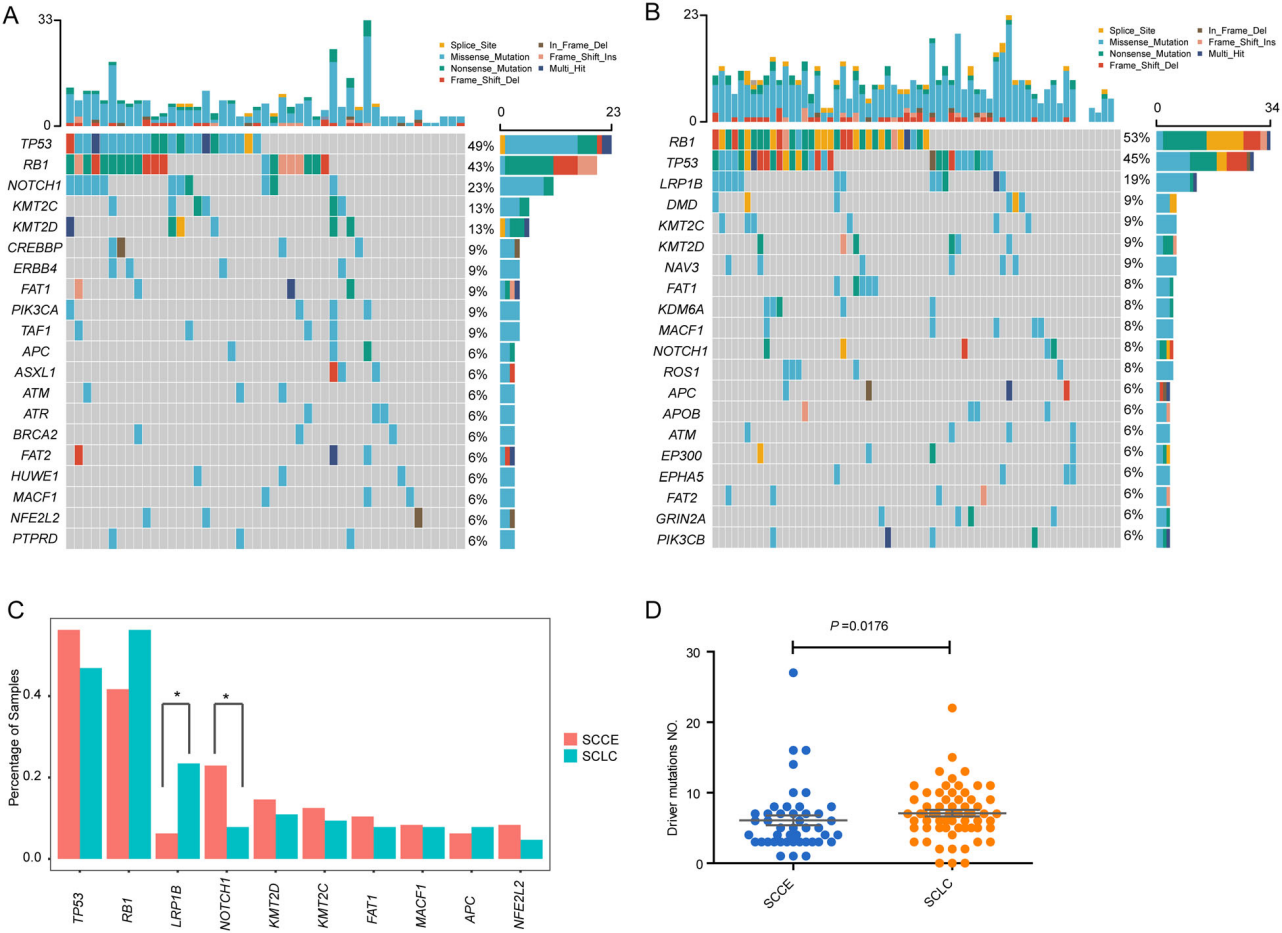
Driver mutation spectrum of SCCE and SCLC. **A** Driver mutation profiling of SCCE (Top20 was shown); **B** Driver mutation profiling of SCLC (Top20 was shown); **C** Comparison of mutation frequency between SCCE and SCLC; **D** Driver gene NO. was significantly lower in SCCE than that in SCLC.

We performed WES sequencing of FFPE samples obtained from 48 SCCE and 64 SCLC patients, with an average sequencing depth of 133× and 157×, respectively (Fig. 1). As shown in Fig. 2, the median number of altered driver oncogenes in the SCCE cohort was five (range, 1–27), while it was seven in the SCLC cohort (range, 0–22). *TP53* and *RB1* were the most frequently mutated genes in both SCCE and SCLC patients, mutated in 60% and 54% patients, respectively, in SCCEs, and 64% and 77% in SCLCs. Genes in *NOTCH* family have been validated as important tumor suppressors and master regulators of neuroendocrine tissue differentiation in SCLC and SCCE^8,9^. Mutations in *NOTCH* family occurred in 25% SCCEs (12/48), higher than in SCLCs (15.62%, 10/64). Comparison of the prevalence of mutations in SCCE and SCLC showed significantly enriched *NOTCH1* mutations in SCCE (two-tailed Fisher’s exact test, *p* = 0.0339), and *LRP1B* mutations in SCLC (two-tailed Fisher’s exact test, *p* = 0.0125). Besides, the number of identified driver mutations was significantly lower in SCCE than that in SCLC (Mann−Whitney test, *p* = 0.0042).

No significant difference in driver mutation number was found between upper and mid-lower SCCE (Mann−Whitney test, *p* = 0.1451, Fig. S1), due to the patient bias (upper, 6%, 3/48; mid-lower, 94%, 45/48).

### Different clonal and subclonal events between SCLC and SCCE

**Fig. 3:**
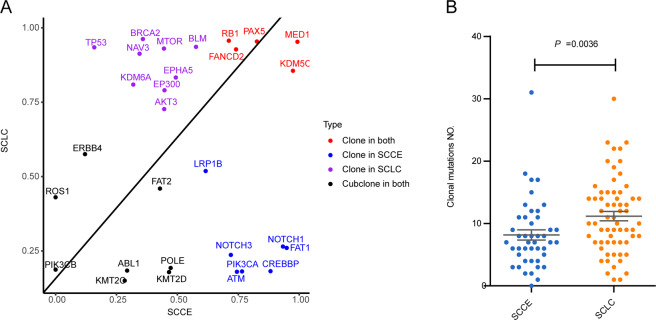
Clonal and subclonal driver mutations in SCCE and SCLC. **A** Clonal and subclonal driver gene in SCCE (*x*-axis) and SCLC (*y*-axis), clonality of genes was identified using PureCN, and presented with different colors; **B** the total number of clonal mutations differed between two cancers, and presented lower in SCCE, compared with SCLC (median, 7 v.s. 10 mutations, Mann−Whitney test, *p* = 0.0036).

To compare the clonal architectures in SCLCs and SCCEs, the cancer cell fraction (CCF) of each driver mutation was calculated and compared between the two cohorts. Clonality analysis of 29 recurrent driver mutations was performed in SCCE and SCLC subsequently. In total,13 of 29 (46%) recurrent driver mutations were evaluated to be clonal events in SCCE, which occurred at an early stage during tumor development, while 16 clonal driver mutations were found in SCLC (Fig. 3A). Among these recurrent mutations, mutations in *RB1*, *PAX5*, *FANCD2*, *MED12*, and *KDM5C* were clonal in both tumors. Notably, mutations in *NOTCH1/3*, *PIK3CA*, and *ATM* were specifically clonal in SCCE, while *TP53* mutations were specifically clonal in SCLC. Mutations in *POLE*, which could lead to the hypermutated and ultramutator phenotypes and possibly improved response to immune therapies in patients, were observed subclonal in both cancers.

Despite the distinct clonalities observed in driver mutations, the total number of clonal mutations also differed between two cancers and presented lower in SCCE compared with SCLC (median, 7 v.s. 10 mutations, Mann−Whitney test, *p* = 0.0036, Fig. 3B). However, the total number of subclonal mutations showed no difference.

### Association between genetic features with prognosis

**Fig. 4:**
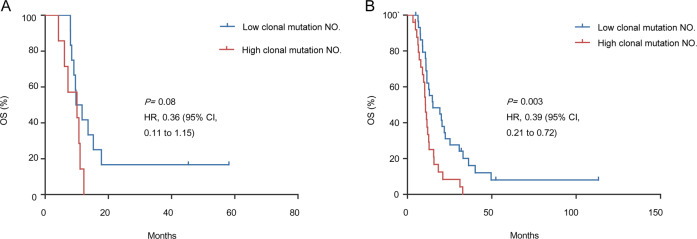
Clonal mutation NO. in relation to the outcome of both SCCE and SCLC. **A** Kaplan−Meier analysis of overall survival (OS) in SCCE patients with more and less clonal mutation NO. (HR, 0.36; 95% CI, 0.11–1.15; *P* = 0.08). **B** Kaplan−Meier analysis of overall survival (OS) in SCLC patients with more and less clonal mutation NO. (HR, 0.39; 95% CI, 0.21–0.72; *P* = 0.003).

We next explored whether these characteristics of clonal events were related to the patient outcome. Median values of clonal mutation number were used as the criteria to define high or low clonal mutation number. As a result, overall survival (OS) was shorter in SCCE patients with higher burdens of clonal mutations (median OS, 10.1 months v.s. median OS, 10.7 months; HR, 0.36; 95% CI, 0.11–1.15; *p* = 0.08; Fig. 4A). This difference in prognosis was also significant in SCLC (HR, 0.39; 95% CI, 0.21–0.72; *p* = 0.003).

**Fig. 5:**
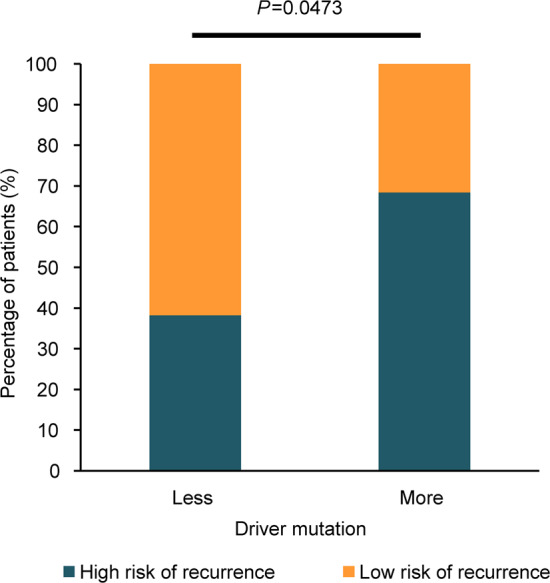
Driver mutation number in relation to the mortality rate of SCLC. SCLC patients with more driver mutation number showed a higher 1-year mortality rate (68%) than those with less driver mutation number (38%, Fisher’s exact test, *P*â€‰=â€‰0.0473).

In SCLC, a higher risk of mortality was observed in patients with more driver mutations (1-year mortality rate: 68% v.s. 38%, Fisher’s exact test, *p* = 0.0473; Fig. 5). In conclusion, a higher mutational burden was associated with the poor prognosis in patients, possibly due to increased tumor malignancy.

## Discussion

This study indicated that SCCE and SCLC exhibited both convergent and distinct mutational and evolutionary characteristics. Previous study profiled the genomic landscape of SCCE and provided evidence to elucidate the origination of SCCE. To our knowledge, for the first time, we compared the driver events between SCCE and SCLC and found that SCCE showed a relatively simple composition of driver mutations. This finding revealed that less driver events were involved in SCCE tumorigenesis and tumor development, and they might play vital roles in tumor cell differentiation and cell migration^10–15^. The low-density lipoprotein receptor-related protein 1B (LRP1B), which encoding endocytic LDL-family receptor, among the top 10 significantly mutated genes in human cancer, has been demonstrated associated with high tumor mutation burden and reported to be a biomarker indicating prolonged survival in melanoma patients and non-small-cell lung cancer patients receiving immunotherapies^16,17^, was found frequently mutated in SCLC, and might also serve as a biomarker to predict response to immune therapies in SCLC. In this regard, SCCE harbored recurrent *NOTCH1* mutations, contributing to the formation of an immune-suppressive tumor microenvironment (TME) and exhibiting great potential in the development of antitumor immunotherapies^18,19^. In the immune system, CD8^+^ T cells can kill tumor cells with cytotoxic molecules, such as granzymes and perforin. An increasing number of reports have demonstrated that the *NOTCH* pathway was required for CD8^+^ T-cell activation and homeostasis; it is therefore probable that the alteration of this pathway could be useful in guiding the treatment decisions of ESCC when the immune system is involved.

More obvious differences were presented in the clonalities of these driver mutations. Although *TP53* was recurrently mutated in both cancers; mutations in this gene were identified subclonal in SCCE but clonal in SCLC. Loss of *TP53* function is well-known to influence cell cycle checkpoint controls and apoptosis, and regulate other key stages of metastatic progression, such as cell migration and invasion. Earlier aberrance in *TP53* gene may lead to more drastic migration and invasion of SCLC^20^. In ESCC and EACC, *TP53* mutations were reported to occur before genome-wide copy number alterations and play an early and crucial role in the tumorigenesis and progression. Even normal esophageal mucosa and esophagitis were reported to harbor *TP53* mutations^21^. However, our study showed subclonal *TP53* mutations of SCCE, which suggest *TP53* mutated relatively later than other clonal genes. This result may distinguish somatic genetic abnormalities in *TP53* that were not necessary for tumorigenesis, but capable of clonal expansion of SCCE.

We also identified SCCE-specific clonal mutations in *PIK3CA*, *NOTCH* family, and *ATM*. *PIK3CA* mutations have been reported to play various roles in tumorigenesis and drug resistance. Especially in breast cancer, activating PI3K mutations may have some general effects upon both proliferation and chemotherapy responses^22^. *ATM* gene and its protein product are critically involved with the DNA damage response (DDR) pathway, which orchestrates the detection and repair of DNA damage with transient cell cycle arrest to ensure maintenance of genomic stability and cell viability. Loss function of *ATM* is associated with the hypersensitivity to ionizing radiation, cancer susceptibility, immunodeficiency, and genomic instability. Carcinoma deficient of *ATM* frequently displays chemotherapy resistance and poor survival^23^. These clonal events suggested diverse phenotypic characteristics and heterogeneous response to systemic therapy^24,25^. On the side, drug development and precision medicine strategies will likely require not only an understanding of cancer genes and mutational processes but also an appreciation of the clonal status of driver events and the timing of mutational processes. Targeted drugs of PI3K pathway and effective immune checkpoint inhibitors that have been approved in other solid tumors may provide clinical benefit for fractional SCCE, especially patients with this clonal gene aberrance^26–28^.

Moreover, in SCCE, patients with less clonal mutations showed improved survival, and this prognostic value of clonal architecture was also verified in the SCLC cohort. In conclusion, tumor heterogeneity results from the occurrence of various driver mutations, and subclonal events that emerged during the process of tumor evolution would affect patients’ prognosis^29^. As previously reported in NSCLC, heterogeneous driver alterations that occurred later in evolution were found in more than 75% of the tumors and were common in *PIK3CA* and *NF1* and in genes that are involved in chromatin modification and DNA damage response and repair. High proportion of subclonal copy-number alterations was associated with an increased risk of recurrence or death^23^. A broad understanding of the heterogeneity of driver genes, deciphering the clonal and subclonal frequencies, and the timing of mutational processes involved in SCCE evolution, is lacking.

Despite the long follow-up time in our study, our conclusions may be affected by the small number of patients and retrospective analysis. Second, whether some clonal events would contribute to the chromosomal instability due to aberrant driver activation, needed to be further explored. Third, re-biopsy samples could provide horizontal temporal information of tumor evolution. Therefore, large-scale prospective studies, multi-omics analysis together with functional exploration and serial biopsy samples, will be required to further elucidate the evolutionary mechanisms and validate prognostic factors of SCCE.

In summary, SCCE showed distinct genetic underpinnings and clonal architectures compared with SCLC. Organ-specific clonal events revealed differences in tumorigenesis and predicted patients’ prognosis, showing great potential to translate into clinical practice to guide disease management and inform treatment decisions. Clonal mutations could serve as a potential biomarker related to the prognosis of both SCCE and SCLC patients.

## Materials and methods

### Patients and samples

Patients with SCCE and SCLC were eligible for this study. The inclusion criteria were as follows: (1) patients with a pathologically confirmed diagnosis of SCCE or SCLC, (2) age ≥ 18 years, (3) Eastern Cooperative Oncology Group performance status of 0−1, (4) without any previous treatment, and (5) provided written informed consent. Tumor samples of 48 SCCE and 64 SCLC patients who were treated at Zhejiang Cancer Hospital, Fujiang Cancer Hospital, and Hebei Cancer Hospital were enrolled in this study. Tumor samples were obtained from resection surgeries or tissue biopsies from August 2009 to August 2017. DNA was isolated from formalin-fixed paraffin-embedded (FFPE) tissue. All patients provided with informed consents and all tumors were reviewed and histopathologically confirmed to be SCCEs or SCLCs. The American Joint Committee on Cancer (AJCC) 7th edition TNM classification of esophageal and lung carcinoma was used for the tumor staging. This study was approved by the institutional review board (IRB) of all hospitals and was conducted in accordance with the Helsinki’s Declaration.

### WES sequencing

In all, 10−15 10 μm-thick FFPE sections were subjected to DNA extraction using DNeasy Blood & Tissue Kit (Qiagen, Hilden, Germany) according to the manufacturer’s protocol. Tumor samples were carefully dissected to avoid contamination with normal parenchymal and stromal tissues. The concentrations of DNA were measured by Qubit fluorometer (Invitrogen, Carlsbad, CA, USA) and the Qubit dsDNA BR (Broad-Range) Assay Kit (Invitrogen, Carlsbad, CA, USA). A total of 1 µg of DNA was fragmented into 200–250bp segments using a Covaris S2 instrument (Woburn, MA, USA). The KAPA DNA Library Preparation Kit (Kapa Biosystems, Wilmington, MA, USA) was used to construct sequencing libraries according to the manufacturer’s protocol and the libraries were hybridized to SeqCap EZ Exome 64 M (Roche NimbleGen, Madison, WI, USA). In brief, the fragments were end-repaired, A-tailed and adapter-ligated, amplified, hybridized to the SeqCap EZ library for 72 h, and then washed. The captured DNA was recovered using Streptavidin Dynabeads (Life Technologies). The captured DNA was then amplified by PCR. The purified captured DNA was then clustered using the cBot (Illumina, San Diego, CA, USA), and sequenced using the HiSeq 3000 Sequencing System (Illumina, San Diego, CA, USA) with 2×101-bp paired-end reads.

### Data processing

Exome sequence data were analyzed employed Sentieon Genomics software (version 201711.05)^30^. Adaptor sequences and low-quality reads that included high proportions of Ns (>10%) and low-quality bases (>50% bases with quality <5) were first filtered out. Eligible sequencing reads were aligned to the human reference genome (build GRCh37) with BWA (version 0.7.12, http://bio-bwa.sourceforge.net/). Sentieon’s quality control algorithms based on Picard’s alignment metrics were performed to calculate the aligned sequencing data. Subsequently, Picard’s CollectHsMetrics was used to collect key metrics of the aligned reads, the average coverage, as well as other metrics. BAM-matcher was utilized to ensure that paired samples came from the same patient by comparing the tumor and adjacent normal tissue BAMs^31^.

### Identification of somatic mutations

Somatic single-nucleotide variations (SNVs) and small insertions/deletions (Indels) were confirmed by Sentieon’s TNsnv. Mutations were filtered as follows to exclude common single-nucleotide polymorphisms (SNPs). Variants with frequencies ≤0.0002 in ExAC (The Exome Aggregation Consortium), ≤0.001 in ESP6500 (The Exome Sequencing Project), ≤0.01 in dbSNP (The Single-Nucleotide Polymorphism database), and 1000 G (The 1000 Genomes Project Consortium) databases were retained. Meanwhile, variants (except hot spots TP53 and RB1) with supported reads ≥5 (3), variant allele fraction ≥5% (3%), and coverage ≥30 in tumors were kept as well. To filter out germline polymorphisms detected in tumor samples without paired control, mutations were furthermore filtered by highly (5% or above) mutated genes in previous research^8,32^ in SCLCs, while a gene list containing highly altered genes in a former study^31^ and genes mutated over two patients in the paired SCCE patients in this cohort was used for mutation filtration in SCCEs with a single-tumor sample. In addition, mutations in genes reported to possibly affect the benefits of immunotherapies were also kept. Driver mutations were ascertained based on a comprehensive analysis of oncogenic driver genes^33^. The candidate variants were all manually verified in the Integrative Genomics Viewer (IGV, http://www.broadinstitute.org/igv/). Nonsynonymous mutations were annotated by ANNOVAR (http://www.openbioinformatics.org/annovar/).

### CCF and clonality analysis

CCF and clonality of detected mutations were analyzed with PureCN software (https://github.com/lima1/PureCN) using default settings^34^.

### Statistical analysis

Mann−Whitney test was employed to compare the median of different groups. The Fisher’s exact test was performed to test the mutational frequencies between groups. The Wilcoxon test and Fisher’s exact test were employed to compare the significant differences between two groups. The cutoff value of clonal mutation number equaled to median of each cohort. Kaplan−Meier survival plots were generated for studying the associations between genomic features with patients’ prognosis using log-rank tests. All statistical analyses were performed with SPSS (v.21.0; STATA, College Station, TX, USA) or GraphPad Prism (v. 6.0; GraphPad Software, La Jolla, CA, USA) software. Statistical significance was defined as a two-sided *p*-value of <0.05.

## Supplementary information

Figure S1

Supplementary Table
