# Supplementary figures and images for: Distinct mutational backgrounds and clonal architectures implicated prognostic discrepancies in small-cell carcinomas of the esophagus and lung
Source: Cell Death Dis. 2021 May 12;12(5):472. doi: 10.1038/s41419-021-03754-0 (PMC8115141; doi:10.1038/s41419-021-03754-0)

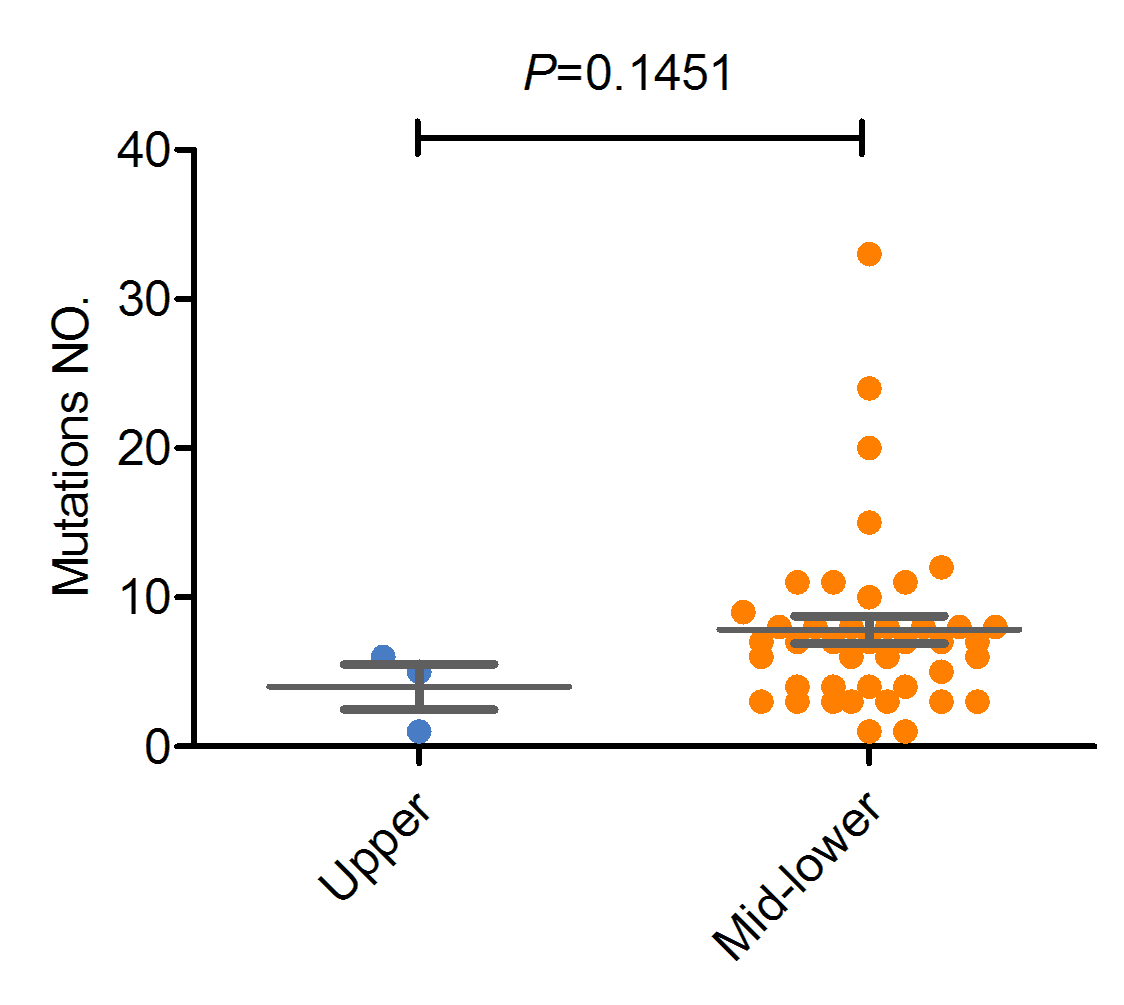

Supplement: Supplementary file 1 — Figure S1 [file 41419_2021_3754_MOESM1_ESM.tif]
